# Botanical Description of British Plants

**Published:** 1806-03

**Authors:** 


### *Botanical Description of British Plants,*

[ Continued from Vol. xv. p. 66—72. ]

3. ARBUTUS. *A. uva ursi.* — *Vaccinia-ursi.*

*Ang.* Bear berries. Bear whortle berries. Bear berry. Strawberry-tree. Trailing Arbutus.

*Gen. Desc.* *As above.*

*Spec. Desc.* *Stem* trailing. *Leaves* oblong-egg-shaped, very entire, veined like net work underneath. *Bloss* flesh-colour; tinged with red, mouth contracted. *Berries* red. *Dry heaths and woods.* BL. May, June.

*Use.* The leaves have in a dried state a bitterish astringent taste. The berries insipid, pulpy, and mealy. The uva-ursi was much employed by the Ancients as an astringent; but in modern practice was neglected, till, about the middle of the 18th century, it acquired celebrity for its efficacy in *calculous* and *nephritic* affections, and also in

---

in almost every complaint to which the urinary organs are liable, as ulcers of the kidneys and bladder, cystirrhœa, diabetes, &c. It was first used at Montpellier for this purpose, and afterwards by Dr. de Haen at Vienna, who relates several cases, in which it proved of the greatest service. But in England its success has been more uncertain; some patients found no relief, and some thought their complaints rather aggravated than alleviated; while in other calculous and nephritic cases, the symptoms have been almost entirely removed. Perhaps, upon the whole, it may be found no better than other vegetable astringents; some of which have been long used by the country people in gravelly complaints and with very great advantage, tho' hitherto unnoticed by the regular practitioners. *Half a drachm* of the powdered leaves given every day, or every other day, has been found useful in calculous cases. From Dr. Alexander's experiments, the leaves seem to possess very little diuretic power; and those made by Murray show that they have no material effect upon the urinary calculi; their efficacy therefore in calculous diseases must be ascribed to their astringency, and this is confirmed by the opinion of Dr. Cullen, *M. M. ii. p.* 12.—The leaves may be employed either in powder or decoction; the former is commonly preferred, and given in doses from a scruple to a drachm two or three times a day.—Those plants which grow in dry, lofty, and exposed situations, are preferred for medical use, to those found in vallies and shady ground.—This plant is much used in Sweden to dye an ash-colour, and to tan leather. *Woodville. Withering.* Horses, cows, sheep and goats refuse it.

4. SCELERANTHUS. *S annuus.*

*Ang.* German knotgrass. Annual knawell.

*Gen. Desc.* Cal. one-leaf; bloss. o, seed one, inclosed in a cup.

*Spec. Desc. Cal. Segments,* thornless, tapering to a point; open, the fruit is ripe. *Branches* opposite, or alternate, woolly. *Leaves* with membranaceous and downy edges, smooth above. *Flowers* in clusters, greenish. *Stamens* 6 to 9, unequal, some barren. *Sandy ground, corn fields.* BL. July, Aug.

*Use.* The Swedes and Germans make use of this plant to cure the *tooth-ach,* receiving into their mouths the vapour arising from a decoction of it.—Sheep and goats eat it; cows refuse it. *Withering.*

Decandria Digynia.

5. Sceleranthus. *S. perennis.*—*Polygonum cocciferum.*

*Ang.* Perennial Knawell. Hoary Knawell.

*Gen. Desc.* As above.

*Spec. Desc.* *Cal. segm.* blunt; nearly closed when the fruit is ripe. *Stems* not always woolly. *Leaves* sea-green, fringed. *Stam.* 10. *Flowers* greenish, edged with white. *Sandy corn fields.* Bl. July, Aug.

*Use.* Upon the roots of this plant is found in the summer months, the Polish cochineal, *Coccus Polonicus,* an insect which affords an excellent and valuable scarlet dye. *Hill.*—*Withering.*

6. Saponaria. *S. officinalis.*—*S. vulgaris.* *Lychnis Saponaria dicta.*

*Ang.* Bruise wort. Soap wort.

*Gen. Desc.* Cal. one-leaf, naked. Petals five, with claws. Caps. oblong, one-cell.

*Spec. Desc.* *Cal.* cylindrical. *Leaves* egg-spear-shaped, sitting, opposite, three-fibred. *Flowers* terminating, flesh-colour, or white. *Meadows, hedges.* Bl. July, Aug.

*Use.* Its medical effects, which must be considerable, have not yet been accurately ascertained. The diseases for which it is recommended, *syphilis, gout, rheumatism,* and *jaundice,* are not perhaps those in which its use may be most availing: for a fancied resemblance of its roots with those of sarsaparilla, seems to have led Physicians to think them similar in their effects; and hence they have both been administered with the same intentions, particularly in fixed pains and venereal affections. Bergius says, "In arthritide, cure mercuriale, &c. nullum aptiorem potum novi." However, according to several writers, the most inveterate cases of *syphilis* were cured by a decoction of this plant, without the use of mercury. *Rudius Septalius, &c.* See *Woodville.* Boerhaave, as we learn from Haller, entertained a high opinion of its efficacy in jaundice, and other visceral obstructions. The whole plant is bitter. A decoction of it applied externally cures the *itch.* The Germans use it instead of sarsaparilla in *venereal* complaints. M. Andry of Paris, cured violent gonorrhœas, by giving daily half an ounce of the inspissated juice. By the use of the extract and a decoction of the leaves and roots, M. Jurine cures old venereal complaints, such as ulcers, pains, and emaciations, which resisted the use of mecury. *See Journ. de. Med. t. lxvi, p.* 478. Bruised and agitated with water it raises a lather like soap, which which washes greasy spots out of cloaths.—*Withering.* It has been used by mendicant Monks for washing their cloaths; and Bergius declares, that it has all the effects of soap itself, but that this saponaceous quality is not injured by acids like that of common soap.—*Woodville.*

Decandria. Trigynia.

7. CUCUBALUS. *C. behen.*—*Lychnis sylvestris. Papaver spumeum.*

*Ang.* Spatling poppy. White bottle. Bladder campion. White ben.

*Gen. Desc.* Cal. inflated. Petals five, with claws, not crowned at the mouth. Caps. three-celled.

*Spec. Desc.* Cal. nearly globular, smooth, white, or purplish, with a net-work of green or purple veins. *Leaves* egg-spear-shaped, glaucous, smooth. *Flowers* white. Pistils, sometimes on the sea-coast 4. *Corn fields, dry meadows, pastures.* BL. June, Aug.

*Use.* By the inhabitants of Gothland the leaves are applied to *erysipelatous* eruptions.—These leaves boiled have something of the flavor of pease; and in 1685, when a swarm of locusts had destroyed the harvest in Minorca, they proved of very considerable service to the inhabitants of that Island.—*Withering.*

8. STELLARIA. *S. media.*—*Alsine media.*

*Ang.* Common chickweed.

*Gen. Desc.* Cal. five-leaves, expanding. Petals five, divided down to the base. Caps. one-celled. Seeds many.

*Spec. Desc. Petals,* deeply divided, white. *Stamens* vary greatly in number. *Leaves* egg-heart-shaped. *Stems* with a hairy ridge on one side. *Caps.* six-valve; *Seeds,* round, compressed, yellow, rough, with little tubercles. *All soils, from the dampest to the driest.* BL. March, Oct.

*Use.* The young shoots and leaves boiled, are a very good eatable; they can scarcely be distinguished from spring spinach, and are equally wholesome.—*Withering.* This species of stellaria is a notable instance of what is called the *sleep of plants;* for every night the leaves approach in pairs, so as to include within their upper surfaces the tender rudiments of the new shoots; and the uppermost pair but one, at the end of the stalk, are furnished with longer leaf-stalks than the others, so that they can close upon the terminating pair, and protect the end of the branch, *Linn.* The flowers are open from nine o'clock till till noon, but of a day that it rains they do not open; after rain they become pendent, but in a few days rise again and are upright. Swine are extremely fond of it; cows and horses eat it; sheep are indifferent to it; goats refuse it. It is a grateful food to small birds, and to young chickens.—*Withering.*

Decandria. Pentagynia.

9. SEDUM. *S. telephium.*

*Ang.* Orpine stonecrop.

*Gen. Desc.* Cal. five-cleft; bloss. 0, or five-pet; nectariferous scales five, at the base of the germens. Caps. five, distinct like a legumen.

*Spec. Desc.* *Leaves* flattish, serrated; *corymbus* leafy; *Stem* upright; *Flowers* white, or more often purple. *Pastures, hedges.* BL. Aug.

*Use.* A decoction of the leaves in milk is a forcible *diuretic.* It has been given with success to cure the *piles.*—Cows, goats, sheep, and swine eat it; horses refuse it. *Linn.*

10. SEDUM. *S. acre.—Sempervivum minus vermiculatum acre. Illecebra minor acris.*

*Ang.* Pepper stonecrop. Wall pepper. Wall stonecrop.

*Gen. Desc.* As above.

*Spec. Desc.* *Leaves* nearly egg-shaped, growing to and sitting, bulging, nearly upright, alternate; *tuft* with three divisions. *Shoots* club.shaped, slosely tiled with leaves on every side. *Flowers* terminating, yellow. *Walls, roofs, rocks, dry pastures.* BL. June, July.

*Use.* It is acrid and biting to the taste. The juice externally applied is recommended in *ulcerous sores* and *cancers;* taken internally, it operates strongly as an *emetic* and *cathartic.* One oz. of it boiled in 12oz. of ale, and taken in four doses, has been found serviceable in the *dropsy.*—*Lightfoot.* This plant, applied externally, blisters; taken internally, it excites vomiting. In *scorbutic cases,* and *quartan agues,* it is an excellent medicine under proper management.—*Withering.* This species of sedum, in its resent state, is extremely acrid like the hydropiper; hence, if taken in large doses, it acts powerfully on the primæ viæ, proving both *emetic* and *cathartic;* applied to the skin, as a cataplasm, it frequently produces vesications and erosions. Boerhaave imagined therefore that its internal employment must be unsafe; but experience has discovered that a decoction of it is not only safe, but of grea efficacy in *scorbutic* complaints; for which purpose a handful of the herb is directed by Dr. Below, of Sweden, to be boiled in eight pints of beer till reduced to four, of which 3 or 4 oz. are to be taken every, or every other morning. (See *Misc. Nat. Cur. Dec.* 1. *Ann.* 6. *Obs.* 22. *p.* 49.) Milk has been found to answer this purpose better than beer; *Lange. Remed. Bruns. Domest. p.* 121. Not only ulcers simply scorbutic, but those of a *scrophulous* and even *cancerous* tendency, have been cured by the use of this plant, of which several instances are related by Marquet, *Mem. Sur. l'Illecebra, &c.* He likewise found it useful as an external application in destroying fungous flesh, and in promoting a discharge in gangrenes and carbuncles. The stopping of *intermittent fevers,* is another effect for which this plant has been esteemed.—*Woodville.* Goats eat it; horses, cows, sheep, and swine refuse it.

11. SEDUM. *S. rupestre.*

*Ang.* Rock stonecrop.

*Gen. Desc. As above.*

*Spec. Desc. Leaves* awl-shaped, tiled in five lines, crowded, loose at the base, tea green. *Flowers* in a close, thick, branched tuft; yellow. *Rocks.* BL. July.

*Use.* Both this and the S. reflexum *(yellow stonecrop, or prick madam,* which differs from this only in the lower leaves being bowed back,) are gathered frequently in Holland and Germany, in order to mix with lettuces in sallads. It is acrid to the taste.

12. OXALIS. *O. acetosella.—Lujula, Alleluja, Acetosella, Oxys alba.*

*Ang.* Wood sorrel. Cuckoo-bread. Sour trefoil.

*Gen. Desc.* Cal. five-leaved. Petals connected by claws. Caps. five-sided, opening at the corners.

*Spec. Desc. Stalk* with one flower. *Leaves* three together, often purple underneath; *leafits* inversely-heart-shaped, hairy; they close against rain. *Flowers* large, white, beautifully veined with purple. *Woods, shady hedges, heaths.* BL. April.

*Use.* Though totally inodorous, this plant has a grateful acid taste, more agreeable than that of common sorrel, (Rumex acetosa) and nearly approaching to that of the juice of lemons or the acid of tartar, with which it also corresponds in a great measure in its medical effects, being esteemed *refrigerant, antiscorbutic* and *diuretic.* It is recommended by Bergius in inflammatory, bilious and putrid fevers. But its principal use is to allay inordinate heat and and to quench thirst; for this purpose a pleasant whey may be made by boiling the plant in milk, which under certain circumstances may be preferable to the conserve directed by the London College, though an extremely grateful and useful medicine. Many have employed the root, probably more on account of its beautiful red colour, than for its superior efficacy.—*Woodville.* An infusion of the leaves is an agreeable liquor in ardent fevers, and boiled with milk, they make a pleasant whey.—*Lewis.* This plant, which is acid and cooling, is recommended in *fevers* and for the *scurvy.* In the Isle of Arran, a whey or tea is made of it, which is used with good success in putrid and other fevers.—*Lightfoot.*

The *conserve* is directed to be made of the leaves, beaten with thrice their weight of fine sugar.—The expressed juice depurated, properly evaporated, and set in a cool place, affords a crystalline acid salt in considerable quantity, which may be used whenever vegetable acids are wanted. It is sold under the name of Essential Salt of Lemons, and is employed to take ink-stains and iron-moulds out of linen.—*Withering.*

It has lately been discovered that the leaves and stalks, wrapped in a cabbage leaf and macerated in warm ashes till reduced to a pulp, are a successful application to *scrophulous* ulcers; this poultice should remain on the sore for twenty-four hours and be repeated four times. Afterwards the ulcer is to be dressed with a poultice made of the roots of the meadow-sweet, bruised, and mixed up with the scum of sour butter-milk. *Beddoes on Fact. Airs.* The grateful acid taste of the leaves render them useful in sallads, where, in some measure, it supplies the place of vinegar.

13. Spergula. *S. arvensis.*

*Ang.* Corn spurry.

*Gen. Desc.* Cal. five-leaved. Pet. five, entire. Caps. egg-shaped, one-celled, five valves.

*Spec. Desc.* *Leaves* in whirls, three on each side, cylindrical, thread like, woolly, clammy. *Stem* thick at the joints. *Fruit stalks* branching. *Flowers* white. *Stamens* 5 to 10. *Seeds* rough, black with a white border. *Corn fields, gravel walks.* Bl. July, Sept.

*Use.* The inhabitants of Finland and Norway, use the seeds to make bread, when their crops of corn fail. Poultry are very fond of them. Experience shews it to be very nutritious to the cattle that eat it.—Horses, sheep, goats, and swine eat it; cows refuse it.—*Withering.*

### Class XI. Dodecandria.

### Dodecandria. Monogynia.

1. Asarum. *A. Europæum.—A. vulgare.*

*Ang.* Asarabacca.

*Gen. Desc.* Cal. three or four cleft, sitting on the germens. Bloss. 0. Caps. leather-crowned, six-celled, no valves.

*Spec. Desc.* *Leaves* kidney shaped, blunt, in pairs. *Flowers* purplish. *Woods,* scarce. Bl. May.

*Use.* The roots and leaves have a nauseous bitterish acrid taste; and they are strongly *emetic* and *cathartic*. The root excited vomiting so constantly that it is proposed by Linnæus as a substitute for ipecacuanha; and Dr. Cullen says, "the root dried only so much as to be powdered, proves, in a moderate dose, a gentle emetic; it will answer commonly in doses of a scruple, sometimes in a less quantity," and "as we judge, may be suited to the purposes of ipecacuanha." In small doses, it is said to be *sudorific, diuretic,* and *emmenagogue, Ray. p.* 108. Spirituous tinctures and watery infusions possess both its emetic and cathartic virtues, but coction in water is said to destroy first the emetic and afterwards the purgative power.—*Ray.* The asarum is at present but seldom given internally, the evacuations expected from its use being procured with more certainty and safety by various other medicines; but is chiefly employed as an *errhine,* or *sternutatory,* and is found to be the most useful and convenient in the Mat. Med. The leaves being less acrid than the root, are preferred for this purpose, by the College; and in moderate doses, not exceeding a few grains snuffed up the nose several successive evenings, produce a pretty large watery discharge, which continues sometimes for several days together, by which head-ach, tooth-ach, ophthalmia, and some paralytic and soporific complaints have been effectually relieved. It is the basis of the pulv. sternutatorius, or pulv. asari compositus. It is observed, *Am. Acad. vii. p.* 307, that when exhibited in a state of *very fine powder,* it uniformly acts as an emetic, but when *coarsely powdered,* it passes the stomach and becomes cathartic.—*Woodville.*

The root powdered, and taken to the amount of 30 or 40 grains, excites vomiting.—The powder of the leaves is the basis of most of the cephalic snuffs, which occasion a considerable discharge of mucus from the nostrils, without much sneezing. An infusion of 1 or 2 drachms of the leaves, in wine, vomits.—Cows eat it.—*Withering.*

Dodecandria. Digynia.

2. CARPINUS. *C. betulus.*

*Ang.* Horn beam tree. Hard beam tree. Horse, or horn beech tree.

*Gen. Desc.* M. and Fem. fl, on the same plant. Bloss. 0. Cal. one-leaf, a fringed scale. M. stamens eighteen or twenty. Fem. germens two, two styles on each, nut-egg-shaped.

*Spec. Desc.* *Scales* of the cones flat. *Bark* smooth, white. *Leaves* oval, pointed, sharply serrated. *Stam.* eight to sixteen. *Woods, hedges.* Bl. May.

*Use.* The inner bark is much used in Scandinavia to dye yellow. The wood burns like a candle; it is very white, very tough, harder than hawthorn, and capable of supporting a great weight; it is useful in turning, and for many implements of husbandry; it makes cogs for mill-wheels, even superior to yew.—Cattle eat the leaves, but pasturage will not flourish in its shade.—It loves a poor stiff soil on the sides of hills; is easily transplanted, and bears lopping.—*Withering.*

3. AGRIMONIA. *A. eupatoria.—Eupatorium veterum.*

*Ang.* Common agrimony.

*Gen. Desc.* Cal. five-toothed, surrounded by another. Petals five. Seeds two, in a caps. at the bottom of the calyx, which becomes indurated.

*Spec. Desc.* *Stem* cylindrical, roughish, hairy. *Stem-leaves* winged, the odd leafit on a leaf-stalk. *Leaves* hairy, covered with rising dots, and segments ending in small reddish glands, interruptedly winged; leafits deeply serrated, oblong-egg-shaped; smallest pair entire. *Fruit* hispid; *fruit-stalks* surrounded at the top with a sort of outer calyx, cloven into five spear-shaped irregular segments, hairy at the edges; within, the fruit-stalks covered with white upright bristles, and above a circle of numerous green awns hooked at the end, and within these the proper calyx of five leaves, spear-shaped, concave, glandular without, within marked with three deeper green lines, ending in a reddish point. *Petals* egg-shaped, concave, slightly notched, twice as long as the cap. *Stam.* five to twelve. *Germen* crowned with the calyx and a yellowish fleshy receptacle. *Styles* thread-shaped. *Summits* two thin lips at the end of each style. Caps. egg-shaped, hairy, ribbed. *Seeds* egg-shaped, flatted on one side. *Flowers* in long, upright, terminating bunches, fine yellow. *Borders of corn fields, hedges, shady places.* Bl. June, July.

*Use.* This plant has been principally regarded in the character of a mild *astringent* and corroborant; and it is recommended by many as a *deobstruent,* especially in *hepatic* and other *visceral obstructions.* Chomell relates two instances of its successful use in cases where the liver was much enlarged and indurated; *usuelles. t. ii. p.* 165. It has been used with advantage in *hæmorrhagic* affections; and to give *tone* to a lax and weak state of the solids. In *cutaneous* disorders, particularly in *scabies,* it is said to manifest great efficacy; *(Becker Diss. de Eupat, &c. Erf.* 1783,*)* for this purpose it was given infused with liquorice in the form of tea: but according to Alston it should be always exhibited in the state of powder.—*Woodville.* An infusion of the root is said to be made use of by the Canadians in burning *fevers,* with great success. An infusion of 6 oz. of the crown of the root, in one quart of boiling water, sweetened with honey, and drank, half a pint three times a day, is said by Dr. Hill, to be an effectual cure for the *jaundice;* he advises to begin with a vomit, afterwards to keep the bowels soluble, and to continue the medicine as long as any symptoms of the disease remain.—*Withering.* The leaves make a very pleasant tea, said to be serviceable in *hæmorrhagies* and in *obstructions of the liver* and *spleen.* They are sometimes used by the country people by way of cataplasms in fresh wounds and contusions.—*Lightfoot.* It has been esteemed a cephalic but now is not used.—*Hill.* The flowers fresh gathered smell like apricots.—Sheep and goats eat it; horses, cows, and swine refuse it.—*Linn.*

( To be continued. )

